# A baseline epidemiological survey for malaria and schistosomiasis reveals an alarming burden in primary schools despite ongoing control in Chikwawa District, southern Malawi

**DOI:** 10.1016/j.crpvbd.2024.100183

**Published:** 2024-05-31

**Authors:** Blessings Chiepa, Rex Mbewe, Michelle C. Stanton, Blessings Kapumba, Eggrey Kambewa, Lucy Kaunga, John Chiphwanya, Themba Mzilahowa, Christopher M. Jones, J. Russell Stothard

**Affiliations:** a Malawi-Liverpool-Wellcome Programme, Blantyre, Malawi; b Liverpool School of Tropical Medicine, Liverpool, L3 5QA, UK; c Malawi University of Business and Applied Science, Blantyre, Malawi; d Neglected Tropical Diseases, Ministry of Health, Lilongwe, Malawi; e Malaria Alert Centre, Kamuzu University of Health Sciences, Blantyre, Malawi

**Keywords:** Malaria, Schistosomiasis, Lower Shire Valley, Child health, Co-infection

## Abstract

Our study rationale was to establish contemporary epidemiological data on malaria and schistosomiasis among school-going children in Chikwawa District before future environmental changes associated with the Shire Valley Transformation Programme occurred. Our cross-sectional surveys tested 1134 children from 21 government-owned primary schools (approximately 50 children per school); rapid diagnostic tests for malaria (Humasis Pf/PAN) and intestinal schistosomiasis (urine-Circulating Cathodic Antigen) were used, with urine reagents strips and egg-filtration with microscopy for urogenital schistosomiasis. All infected children were treated with an appropriate dose of Lonart® (for malaria) and/or Cesol® (for schistosomiasis). Across 21 schools the overall prevalence was 9.7% (95% CI: 8.8–10.6%) for malaria, 1.9% (95% CI: 1.4–2.3%) for intestinal schistosomiasis, and 35.0% (95% CI: 33.6–36.5%) for egg-patent urogenital schistosomiasis. The prevalence of co-infection of malaria with urogenital schistosomiasis was 5.5% (95% CI: 4.8–6.2%). In a third of the schools, the prevalence of malaria and urogenital schistosomiasis was above national averages of 10.5% and 40–50%, respectively, with two schools having maxima of 36.8% and 84.5%, respectively. Set against a background of ongoing control, our study has revealed an alarming burden of malaria and schistosomiasis in southern Malawi. These findings call for an immediate mitigating response that significantly bolsters current control interventions to better safeguard children's future health.

## Highlights

• Overall prevalence of malaria across 21 schools was 9.7%, with a prevalence ≥ 10% in 7 schools and a maximum of 36.8%.
• Overall prevalence of intestinal schistosomiasis was 1.9%, with one school having a prevalence > 10%.
• Overall prevalence of urogenital schistosomiasis was 35.0%, 6 schools had a prevalence ≥ 50%, with a maximum of 84.5%.
• Co-infection was more prevalent in schools to the West of the district though this spatial pattern was heterogeneous.

## Graphical abstract

**Figure:**
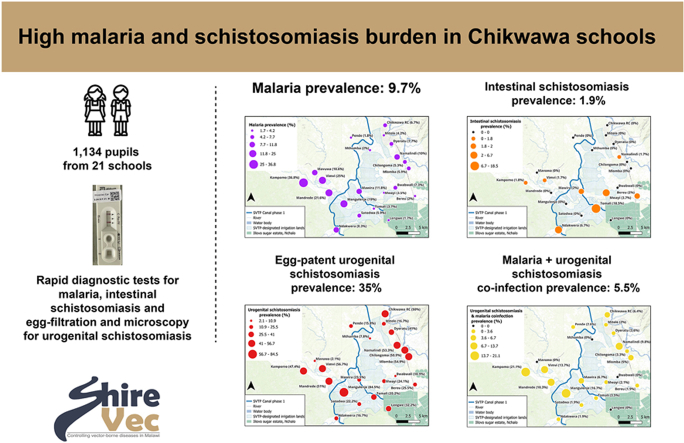

## 1 Introduction

Malaria and schistosomiasis present considerable disease burdens in tropical and sub-tropical countries, such as Malawi (Dassah et al., 2023). Here, malaria national prevalence stands at 10.5% in 6–59-month-olds tested using rapid diagnostic tests and blood smears (Mategula et al., 2023). Before the establishment of a national schistosomiasis control programme, schistosomiasis ranged up to 94.9% for urogenital schistosomiasis and 67.0% for intestinal schistosomiasis (Makaula et al., 2022). Malaria burden is greater in the lakeshore and lower Shire regions. For example, Chikwawa District is burdened by malaria and schistosomiasis. Mategula et al. (2023) classified the district as a moderate burden under the nationwide malaria burden stratification. A schistosomiasis study conducted in the Chikwawa District by Poole et al. (2014) revealed urogenital schistosomiasis prevalence of 17.7% and 45.1% by egg patent urine filtration among 208 pre-school age children and 165 mothers respectively; however, the prevalence of malaria was not assessed in this study.

More broadly, there are gaps in surveillance for malaria and schistosomiasis as health surveys do not concurrently measure each disease simultaneously. Nevertheless, the goal of the Malawi Government is to eliminate malaria by 2030 and reduce the burden of schistosomiasis (Malawi Ministry of Health, 2018; Makaula et al., 2022; Mangani et al., 2022). Various control programmes are being implemented at the national level to achieve these goals. For malaria, the National Malaria Control Programme (NMCP) distributes long-lasting insecticide-treated bednets (LLINs), conducts indoor residual spraying (IRS), and facilitates prompt access to artemisinin-based combination therapy (ACT) and preventive treatment during pregnancy (Mangani et al., 2022; Stanley et al., 2023). In 2019, Malawi also started piloting RTS, S/ASO1 malaria vaccine in 11 districts including Chikwawa (Stanley et al., 2023). In terms of schistosomiasis, the mass drug administration (MDA) of praziquantel is one of the control measures that has been widely carried out annually since 2009 (Ministry of Health and Population, 2018) though COVID-19 restrictions have impacted activities.

Chikwawa District is known for its large-scale Illovo sugar estate occupying 20,925 ha of irrigated lands (Illovo Sugar Africa, 2017) and its considerable livestock farming (Malawi Government, 2020). Both large-scale irrigated agriculture farming and interactions of livestock and humans have been shown to impact the prevalence of malaria and schistosomiasis respectively (Steinmann et al., 2006; Rohr et al., 2019; Jones et al., 2023). In 2019, the Malawi government with funding from the World Bank and other development partners initiated a 14-year programme (2018–2031), the Shire Valley Transformation Programme (SVTP). SVTP will construct a new primary irrigation canal of some 133 km in length that will pass through Chikwawa and put over 40,000 ha of land under irrigation farming (Malawi Government, 2023). This enormous transformation of land will potentially change the distribution of vectors and endemicity of vector-borne diseases like malaria and schistosomiasis in the district (Jones et al., 2023). To understand this interaction between agricultural development and vector-borne disease, a research project, The Shire Valley Vector Control Project (Shire-Vec), will gather empirical data on vector population dynamics and vector-borne disease risk to help design and implement innovative ways of managing vectors in agricultural areas. This will hope to better inform policy directions on controlling vectors in irrigation projects.

Under the umbrella of Shire-Vec, this study sought to establish a contemporary epidemiological baseline for malaria and schistosomiasis. It is envisioned that once the irrigation starts, these data will be vital for future comparisons and for providing evidence-based insights into the changing status of malaria and schistosomiasis in the region.

## 2 Materials and methods

### 2.1 Study site and population

**Fig. 1:**
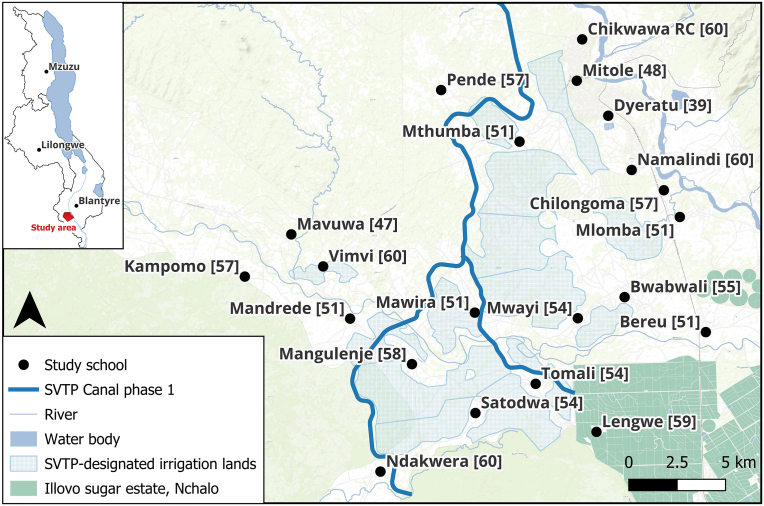
Map of the study area showing the total number of participants across the 21 sample schools. The inset map on the top left corner shows the study location within Malawi.

A cross-sectional study was conducted in 21 government-owned primary schools within three km of the SVTP Irrigation Canal Phase 1 and designated irrigated lands between September and October 2023. In each school, the study team aimed to recruit 25 boys and 25 girls between the ages of 8 and 12 years (inclusive). These were randomly selected at each school using school registers and were given study information leaflets to give to their parents/guardians. Parents were asked to come to school with their children on the day of the survey for consenting purposes. According to the most recent census data (2018), 15.2% of the population in the rural parts of the Southern Region of Malawi are aged 8–12 (Government of Malawi, 2019). Using remotely sensed data for 2020 (CIESIN, 2020), we estimated the population of our study area to be 57,000 of which 8664 (15.2%) will be within the 8–12 age group. We did not have any access or knowledge of recent schistosomiasis surveys for the area; however, we anticipated moderate-to-high levels of endemicity. Assuming a 50% prevalence, a sample of 1050 8–12-year-olds (∼50 per school) out of a total population of 8664 (12.1%) allowed us to estimate the overall prevalence of the study area with a precision of 2.8% at the 95% confidence level. The 50% was a conservative estimate to ensure that the sample size was sufficient for both schistosomiasis and malaria. Body temperature (>38 °C), as measured using handheld infrared thermometers at study enrollment, was the only other exclusion criterion that instigated referral to a local health facility. The distribution of study participants in the study area is depicted in Fig. 1.

### 2.2 Procedures

Participants were tested for malaria and both forms of schistosomiasis on site. Malaria testing was conducted through rapid diagnostic tests (RDT) from finger-prick blood using the Humasis Malaria Pf/PAN Antigen Test (Launch Diagnostics, Kent, UK). This dipstick detects histidine-rich protein 2 (HRP-2) antigen of *Plasmodium falciparum* and common *Plasmodium* lactate dehydrogenase (pLDH), the latter enables detection of non-*P. falciparum* species. Testing of schistosomiasis was two-fold; at each school, participants were asked to provide urine samples, and these were tested for intestinal schistosomiasis using the urine-Circulating Cathodic Antigen (CCA) lateral flow test (Rapid Medical Diagnostics, Pretoria, South Africa) and microhematuria for urogenital schistosomiasis by reagent dipstick (Multistix, Siemens, Manchester, UK). Visual haematuria was also noted. To confirm the detection of urogenital schistosomiasis, urine samples were processed at a field laboratory in Chikwawa, where 10 ml was filtered across a 25-μm pore-sized circular nylon filter (1.3 cm in diameter). Filters were then inspected by microscopy ( × 100 magnification) according to World Health Organization (WHO) protocols with eggs classified as absent, less than 10, 11–49, and 50 or more (Genchi et al., 2019).

### 2.3 Data collection

Data relating to the results of the malaria test and urine testing were reported using Open Data Kit (ODK) Collect, an electronic mobile data collection application, on tablet computers. Malaria and schistosomiasis results were also recorded using physical paper copies (laboratory testing treatment lists). Additionally, enrolled children were asked a questionnaire about their demographics, health-seeking behaviour, bednet usage, water contact, and past malaria and schistosomiasis diagnosis and treatment. This questionnaire was administered using ODK Collect. At the end of the survey, all collected data were uploaded on a secure ODK Central server hosted by the Malawi-Liverpool Wellcome (MLW) Programme in Blantyre.

### 2.4 Statistical analysis

After collection, data were inspected first *via* ODK Central and then downloaded offline and cleaned using Microsoft Excel and R Statistical Software version 4.3.0 (R Core Team, 2021). Descriptive statistics and cross-tabulation were computed using R and maps were created using QGIS version 3.36.0-Maidenhead.

### 2.5 Ethical considerations

The study was approved by the College of Medicineʼs Research Ethics Committee (Protocol number: P.03/23/4041) and the Liverpool School of Tropical Medicine Research Ethics Committee (Protocol number: 22–039). Additionally, community engagement was conducted at both the schools and within villages surrounding the schools, with participants inclusive of headteachers as well as Parents-Teachers Committees. Before the survey, a written informed consent process was completed with the parents/legal guardians of all participating children following the College of Medicineʼs Research Ethics Committee and Malawi Liverpool Wellcome Research Programmeʼs Clinical Research Support Unit guidelines. This ensured they were fully informed about the study objectives, procedures, and potential risks and benefits before providing their consent for their childʼs participation. All infected children were treated on-site for malaria with Lonart® and for schistosomiasis with Cesol® by the project nurse and local health surveillance assistant.

## 3 Results

### 3.1 Demographics and characteristics of study participants

A total of 1134 school-going children between the ages of 8 and 12 years were enrolled in the survey across 21 primary schools, against an intended 1050 (50 per school). This represents 108% of study enrollment.

Table 1 indicates the demographics and characteristics of these participants. The gender of the participants was approximately equal with 51.1% (580/1134) male and 48.9% (554/1134) female. There were more older children [12 years old (31.3%); 11 years old (21.9%); and 10 years old (20.4%)] than younger ones [8 years old (12.4%); and 9 years old (14.0%)]. To understand the usage of malaria control strategies, we asked about bednet ownership and usage. In terms of usage, we asked if participants had slept under a bednet the previous night as we assumed that last night was easy to remember and as per standard malaria indicator surveys. Bednet ownership was 81.4% (923/1134), the national average is 82% and 60.4% of the participants reported sleeping under a bednet the night before the study day, the national average for the same is 55% (National Malaria Control Programme (NMCP) and ICF, 2018). Out of all participants, 98.2% (1114/1134) have been tested for malaria with 95% (1077/1134) having been treated for the same and 73.8% (837/1134) had been treated for schistosomiasis previously. About half of the participants (603, 53.2%) played in or around water bodies at or near home whilst only 4% (45) played or interacted with water at school. The “Other type of waterbody” option asked respondents to provide other types of water bodies that were not pre-listed on the questionnaire. According to the free texts’ entry, this response comprised various small-sized permanent to semi-permanent water bodies such as water between farm ridges and fallow, that are called different names in local languages. Out of 105 “Other type of water body at home” were “Canal” (53.3%), “Fallow” (14.3%), “*Thamanda*” (10.5%), “*Thawale*” (8.6%), Mwanza River (7.6%), Well (1.9%), and “*Zithaphwi*” (1.9%). The “Other type of water body at school” were “*Thawale*” (50%), “*Thamanda*” (40.5%), “Rainwater” (4.8%) and “Canal” (4.8%). The terms “*Thamanda*” and “*Thawale*” describe water in between agricultural ridges whilst “*Zithaphwi*” means stationary muddy water including swamps.

**Table 1:** Demographics and characteristics of study participants.

Variable	Response	*N*	%
Sex	Female	554	48.9
	Male	580	51.1
Age (years)	8	141	12.4
	9	159	14.0
	10	231	20.4
	11	248	21.9
	12	355	31.3
Self-assessed health	Fine	1003	88.4
	Not fine	131	11.6
Bednet ownership	Yes	923	81.4
	No	211	18.6
Slept under bednet last night	Yes	685	60.4
	No bednet	211	18.6
	No	124	10.9
	No response	114	10.1
Previously taken a malaria test	Yes	1114	98.2
	No	20	1.8
Treated for malaria previously	Yes	1077	95.0
	No	57	5.0
Previous schistosomiasis treatment	Yes	837	73.8
	No	297	26.2
Play or wash in ponds, rivers, etc. near home	Yes	603	53.2
	No	531	46.8
Play or wash in ponds, rivers, etc. at school	No	1089	96.0
	Yes	45	4.0
Type of waterbody at home	NA	531	46.8
	River	442	39.0
	Other	105	9.3
	Dam	50	4.4
	Pond	6	0.5
Type of waterbody at school	NA	1089	96.0
	Other	42	3.7
	River	3	0.3
Total		1134	100

### 3.2 Prevalence of malaria and schistosomiasis

The prevalences of malaria, intestinal schistosomiasis, and urogenital schistosomiasis across the 21 schools surveyed in Chikwawa are provided in Table 2. The overall malaria prevalence across 21 schools was at 9.7% (110/1; 95% confidence interval, CI: 8.80–10.60%), with Kampopo school, situated furthest away from the Shire River (27 km), reporting the highest prevalence of 36.8% (21/57). Intestinal schistosomiasis had an overall prevalence of 1.9% (21/1134; 95% CI: 1.44–2.26%). Tomali school, located close to Lengwe National Park, had the highest prevalence at 18.5% (10/54) with over 50% (13) of the surveyed schools having a prevalence of 0%. The overall prevalence for urogenital schistosomiasis was 35.0% (397/1134; 95% CI: 33.56–36.46%) with six schools having a prevalence of 50% or more. Mavuwa, another school further from Shire River (24 km) had the lowest prevalence of all the schools at 2.1% (1/47).

**Table 2:** Malaria and schistosomiasis prevalence per school.

School name	Malaria prevalence		Intestinal schistosomiasis prevalence		Urogenital schistosomiasis prevalence		Malaria + urogenital schistosomiasis co-infection prevalence		Total no. of participants	Total no. of school enrolment
	*n*	%	*n*	%	*n*	%	*n*	%	*n*	*n*
Bereu	1	2.0	1	2.0	13	25.5	1	1.9	51	3500
Bwabwali DP	4	7.3	0	0	6	10.9	0	0	55	1693
Chikwawa RC	4	6.7	0	0	30	50.0	3	6.4	60	1269
Chilongoma	3	5.3	0	0	29	50.9	2	3.3	57	633
Dyeratu	3	7.7	0	0	16	41.0	2	3.6	39	2467
Kampomo FP	21	36.8	1	1.8	27	47.4	12	21.1	57	910
Lengwe LEA	1	1.7	0	0	19	32.2	0	0	59	804
Mandrade	11	21.6	0	0	26	51.0	6	10.3	51	876
Mangulenje	11	19.0	0	0	49	84.5	10	16.7	58	1222
Mavuwa	5	10.6	0	0	1	2.1	0	0	47	652
Mawira FP	6	11.8	1	2.0	12	23.5	4	6.7	51	1573
Mitole LEA	2	4.2	0	0	8	16.7	1	2.0	48	1847
Mlomba FP	3	5.9	0	0	28	54.9	3	5.0	51	1493
Mthumba	1	2.0	0	0	4	7.8	0	0	51	1219
Mwayi	2	3.5	2	3.7	13	24.1	1	2.1	54	1149
Namalindi	6	10.0	1	1.7	32	53.3	5	9.8	60	986
Ndakwera	5	8.3	4	6.7	10	16.7	1	1.9	60	1246
Pende	1	1.8	0	0	9	15.8	1	2.6	57	676
Satodwa	3	5.9	0	0	12	22.2	1	1.9	54	1202
Tomali	2	3.7	10	18.5	19	35.2	2	3.5	54	1664
Vimvi	15	25.0	1	1.7	34	56.7	7	13.7	60	718
Total	110	9.7	21	1.9	397	35.0	62	5.5	1134	27,799

*Abbreviation*: *n*, number of cases.

### 3.3 Malaria and schistosomiasis co-infection

Our study also sought to understand the co-infection of malaria and schistosomiasis. This is important as it can help in implementing public health strategies that deal with both diseases simultaneously; to isolate groups of people who are at high risk of both diseases and the presence of both infections usually presents complexities in the diagnosis and treatment of the diseases. Our understanding of these co-infections can have a significant impact on our overall understanding of vector-borne diseases’ interactions in changing landscapes.

Our results show that 5.5% (62/1134; 95% CI: 4.77–6.16%) of the participants had a co-infection of malaria and urogenital schistosomiasis with Kampomo school having the highest co-infection of 21.1% (12/57); co-infection did not occur in four schools. Only 2 participants (*n* = 1134) had a co-infection of intestinal schistosomiasis and malaria.

### 3.4 Schistosomiasis infection intensity

Schistosomiasis infection egg-intensity was observed alongside macro- and micro-haematuria. Results of both egg-intensity and haematuria are provided in Table 3. The infection intensity of egg-patent infection and heavy egg intensity (> 50) was relatively raised. We recorded 96 participants (18 of these were from Mangulenje primary school) with a schistosome egg count of more than 50, and 117 participants (*n* = 1134) with 10–50 eggs.

**Table 3:** Haematuria results showing visible haematuria in urine as “yes” and not observed as “no”. Parasite density (eggs/10 mL urine) as observed by microscopy for urogenital schistosomiasis is reported according to the number of eggs visible.

School name	Visual haematuria		Parasite density (eggs/10 ml urine)			
	No	Yes	0	1–10	10–50	50+
Bereu	44	7	38	6	2	5
Bwabwali DP	54	1	49	4	2	0
Chikwawa RC	58	2	30	9	12	9
Chilongoma	49	8	28	17	5	7
Dyeratu	32	7	23	7	4	5
Kampomo FP	56	1	30	14	10	3
Lengwe LEA	58	1	40	13	4	2
Mandrade	43	8	25	11	8	7
Mangulenje	40	18	9	17	14	18
Mavuwa	47	0	46	1	0	0
Mawira FP	51	0	39	10	2	0
Mitole LEA	47	1	40	3	3	2
Mlomba FP	47	3	23	4	16	8
Mthumba	48	3	47	2	1	1
Mwayi	53	1	41	6	3	4
Namalindi	53	7	28	19	6	7
Ndakwera	60	0	50	6	3	1
Pende	57	0	48	4	3	2
Satodwa	50	4	42	4	5	3
Tomali	52	2	35	14	2	3
Vimvi	57	3	26	13	12	9
Total	1056	77	737	184	117	96

### 3.5 Spatial distribution of malaria and schistosomiasis

**Fig. 2:**
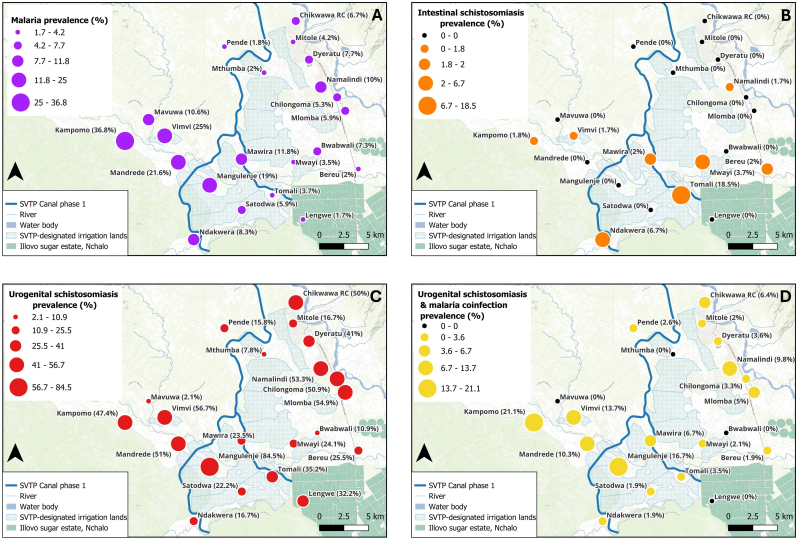
**A** Malaria prevalence. **B** Intestinal schistosomiasis prevalence. **C** Urogenital schistosomiasis prevalence. **D** Malaria and urogenital schistosomiasis co-infection prevalence.

Malaria and schistosomiasis results were mapped to ascertain the spatial distribution of incidence. Fig. 2 shows the distributions of disease by school prevalence.

Malaria prevalence was elevated in the western part of the study area (Fig. 2A) with Kampopo, Vimvi, Mandrede and Mangulenje schools having prevalences of 36.8% (21/57), 25% (15/60), 21.6% (11/51) and 19.0% (11/58), respectively. Intestinal schistosomiasis was clustered in the south specifically along the Mwanza River (Fig. 2B), with the highest record of 18.5% (10/54) at Tomali school located on the path for the main SVPT irrigation canal. There were no intestinal schistosomiasis cases in the north and along the Shire River except for Namalindi primary school with a 1.7% (1/60) prevalence. The highest prevalence of urogenital schistosomiasis was concentrated in schools situated along the Shire River to the northeast and southwest (Fig. 2C). Malaria and urogenital schistosomiasis co-infection were observed in the southwest of the study area (Fig. 2D).

## 4 Discussion

Our study undertook a contemporary baseline epidemiological survey for malaria and schistosomiasis in primary schools in Chikwawa District, Malawi. Both malaria and schistosomiasis remain alarmingly high. We consider these levels to pose a current and future serious public health problem locally. Moreover, our surveillance comes at a time when the Chikwawa District is undergoing rapid landscape changes due to ongoing irrigation canal construction (Malawi Government, 2023), alongside wider climate change (Otto et al., 2022). Indeed, although various control programmes have been initiated and were ongoing during our study, their direct impact on infection control is not sufficient. Understanding vector-borne disease dynamics in Chikwawa before the full future impact(s) of ongoing environmental changes is vital to better tailor future mitigation strategies. This study therefore provides the necessary epidemiological foundation for understanding vector-borne disease dynamics in the face of changing landscapes.

Our study found that the general malaria prevalence across 21 schools was 9.7% (110 out of 1134) with seven out of 21 schools reporting a prevalence equal to or greater than 10%. In terms of recent malaria control activities, there has been IRS in Malawi between 2018 and 2023 in four districts: Nkhata Bay, Nkhotakota, Balaka and Mangochi. However, in Chikwawa District, IRS is localized to the Illovo Sugar Estate (Hoek Spaans et al., 2024). Our study area comprises schools within the Illovo Nchalo sugar estate where IRS has been carried out annually. It was therefore not surprising that Lengwe primary school which is within the estate had only 1 malaria incident out of 59 (1.7%) and two other primary schools close to the estate namely Tomali and Bereu had an equally low prevalence of 3.7% (2 out of 54) and 2.0% (1 out of 51), respectively. It is also important to note that the last ITN mass distribution was conducted in 2021. However, as Chikwawa has been affected by floods and cyclones, various humanitarian organizations have distributed ITNs in affected areas though this net coverage was not reported precisely. Additionally, in 2019, Chikwawa District started implementing the RTS, S/AS01 malaria vaccine to under-fives. Although this may have no direct impact on our study, this vaccine programme is likely to impact the future epidemiology of malaria within the area. Our findings are also concerning considering that they took place during the dry season (September–October) when infection prevalence may not be at its annual maximum. Prevalence is likely to be higher, and perhaps less heterogeneous, during the wet season. We plan to repeat our survey during the next wet season to account for such seasonality. Although malaria prevalence rates have been declining in Malawi (Mategula et al., 2023) and the rest of East Africa region (WHO, 2023), our findings signify that these declines have stagnated and there is more work to be done to reduce the malaria burden to acceptable levels.

The prevalence for urogenital schistosomiasis was 35.0% (397 out of 1134) overall with six schools having prevalences of equal to or greater than 50%. Mangulenje primary school was particularly alarming, having a prevalence of 84.5% (49/58). Field observations also recorded a higher number of participants with advanced schistosomiasis as seen by visual blood in the urine (macro-haematuria) (Table 3). Urogenital schistosomiasis was concentrated in schools along the Shire River and Mwanza River. Furthermore, 442 out of the 1134 participants (39%) reported playing around rivers. Studies have shown that children who regularly swim in rivers are almost 10 times more likely to be infected with schistosomiasis (Ansha et al., 2020). Therefore, the high prevalence of urogenital schistosomiasis in schools along the Shire and Mwanza Rivers is expected. Similarly, the high prevalence of malaria and urogenital schistosomiasis co-infection was higher in schools with a higher prevalence of malaria and urogenital schistosomiasis as expected.

The overall prevalence of intestinal schistosomiasis was 1.9% (21 out of 1134) with one school having a prevalence greater than 10%. Although this is a low prevalence, an emergence of intestinal schistosomiasis alongside a high prevalence of urogenital schistosomiasis is a concern that could accelerate into an outbreak as seen in a study along Lake Malawi (Kayuni et al., 2020). The transmission of intestinal schistosomiasis locally is important when linked to recent findings of *Biomphalaria pfeifferi*, the intermediate host of intestinal schistosomiasis in Malawi (Alharbi et al., 2019), within the Lower Shire Valley, since May 2023 (Russell Stothard, personal observations). Autochthonous transmission of intestinal schistosomiasis here in Chikwawa is now likely, closely tied to locations where this nuisance snail is present. We strongly encourage further efforts in malacological surveillance and future attempts to remove this snail locally.

In terms of spatial patterning, malaria, and schistosomiasis prevalences were heterogeneous by school. However, a more detailed analysis will follow, particularly incorporating surveys done during the wet season. Schools in the western part of the study area had a high prevalence of malaria (Fig. 2). These schools (Kampomo, Vimvi and Mandrede) are also located furthest from public health facilities. It is therefore possible that communities around the schools are reluctant to visit health facilities until they are critically ill due to the distance factor. The undetected malaria cases therefore become reservoirs for further transmissions thus leading to increased malaria incidents. It is also important to note that Tomali and Mangulenje schools with the highest prevalences of intestinal and urogenital schistosomiasis are located very close to the future SVTP irrigation canal which is under construction and where *B. pfeifferi* has been confirmed. It would be important therefore to monitor how these infection dynamics change once the irrigation canal is fully implemented.

In the wake of our findings, we recommend that national control programmes reflect on how their programmes are implemented. Whilst malaria control programmes seem to be effective, we support the targeted intervention approach. For instance, there is a need to develop malaria elimination programmes that deliberately target school-going children and primary schools with a high prevalence of malaria. On the other hand, we recommend that authorities investigate Mangulanje school to determine the transmission source of urogenital schistosomiasis. We call for intensified malacological studies to locate infected snails that may be responsible for both intestinal and urogenital schistosomiasis in Chikwawa and their effective control such as better water management and exploring the application of focal chemical molluscicides. In the longer term, we recommend intensifying MDA to at least twice a year and ensuring treatment coverage of more than 90% is achieved. Most importantly, there is a need for continuous surveillance of malaria, schistosomiasis, and their vectors in the Chikwawa District to monitor how they are impacted by ongoing changes in the landscapes by the SVTP.

## 5 Conclusions

Despite ongoing control, our study reveals an alarming prevalence of malaria and schistosomiasis in Chikwawa District. Our data provide contemporary evidence for health policymakers to reflect on how control programmes are being implemented and, looking to the future, how best to monitor landscape changes caused by large-scale irrigation projects and climate change. For example, there is a clear need to introduce integrated control programmes that target school-aged children and recommend further follow-up studies to investigate seasonality effects.

## Funding

This article is funded by the 10.13039/501100000272NIHR [10.13039/501100000272NIHR 10.13039/100006090Global Health Research Group on Controlling Vector Borne Diseases in Emerging Agricultural Systems in Malawi], grant number NIHR133144. The views expressed are those of the author(s) and not necessarily those of the 10.13039/100012411NIHR.

## Ethical approval

The study was approved by the College of Medicineʼs Research Ethics Committee (Protocol number: P.03/23/4041) and the Liverpool School of Tropical Medicine Research Ethics Committee (Protocol number: 22–039). Additionally, community engagement was conducted at both the schools and within villages surrounding the schools, with participants inclusive of headteachers as well as Parents-Teachers Committees. Before the survey, a written informed consent process was completed with the parents/legal guardians of all participating children following the College of Medicineʼs Research Ethics Committee and Malawi Liverpool Wellcome Research Programmeʼs Clinical Research Support Unit guidelines. This ensured they were fully informed about the study objectives, procedures, and potential risks and benefits before providing their consent for their childʼs participation. All infected children were treated on-site for malaria with Lonart® and for schistosomiasis with Cesol® by the project nurse and local health surveillance assistant.

## CRediT authorship contribution statement

**Blessings Chiepa:** Conceptualization, Methodology, Formal analysis, Investigation, Writing – original draft, Visualization. **Rex Mbewe:** Conceptualization, Methodology, Investigation, Writing – review & editing. **Michelle C. Stanton:** Conceptualization, Writing – review & editing, Supervision, Funding acquisition. **Blessings Kapumba:** Investigation. **Eggrey Kambewa:** Investigation. **Lucy Kaunga:** Investigation. **John Chiphwanya:** Writing – review & editing. **Themba Mzilahowa:** Writing – review & editing, Supervision, Funding acquisition. **Christopher M. Jones:** Writing – review & editing, Supervision, Funding acquisition. **J. Russell Stothard:** Conceptualization, Methodology, Investigation, Resources, Writing – review & editing, Supervision, Funding acquisition.

## Declaration of competing interests

The authors declare that they have no known competing financial interests or personal relationships that could have appeared to influence the work reported in this paper.

## Data Availability

The data supporting the conclusions of this article are included within the article. Raw data can be available upon a reasonable request to the corresponding author.
